# Multi-robot coordination for underwater Mothership–Passenger systems

**DOI:** 10.3389/frobt.2026.1799672

**Published:** 2026-05-18

**Authors:** Nathan L. Butler, Geoffrey A. Hollinger, Xi Yu, Prajjwal Dutta, Atsuhiro Muto, Jessica C. Garwood

**Affiliations:** 1 Collaborative Robotics and Intelligent Systems (CoRIS) Institute, Oregon State University, Corvallis, OR, United States; 2 School of Manufacturing Systems and Networks, Arizona State University, Tempe, AZ, United States; 3 Department of Earth and Environmental Science, Temple University, Philadelphia, PA, United States; 4 College of Earth, Ocean, and Atmospheric Sciences, Oregon State University, Corvallis, OR, United States

**Keywords:** marine robotics, multi-robot coordination, orienteering, reinforcement learning, under-ice exploration

## Abstract

Multi-robot coordination under communication constraints is a fundamental challenge in autonomous systems, particularly in underwater environments, where low-bandwidth acoustic links restrict centralized planning and limit decentralized information propagation. In this study, we explore a “Mothership–passenger” paradigm, in which a capable underwater vehicle deploys and coordinates many lower-cost autonomous robots, enabling broad spatial coverage while reducing the mission risk and cost. We present two algorithms that tightly couple hierarchical guidance with decentralized planning to improve coordination efficiency under these constraints. The first algorithm integrates centralized and decentralized solutions to an underwater multi-robot orienteering problem, enabling globally coherent yet locally adaptive coordination among robots operating under stochastic travel costs and mission disruptions. The second algorithm introduces a learned policy on a Mothership vehicle that adaptively configures individual robot priorities, allowing globally informed guidance to shape local planning under communication and resource constraints. Through simulation and field experiments at an inland lake, we demonstrate that these contributions significantly improve coordination efficiency and robustness compared to fixed-behavior and fully decentralized baselines. These advances are directly motivated by the long-term goal of deploying coordinated robotic teams in isolated environments such as under-ice ocean regions, where the coordination and communication constraints studied here are most acute.

## 1 Introduction

Under-ice ocean environments play a critical role in Earth’s climate and support unique ecosystems, yet they are extremely challenging to observe. These environments include regions beneath sea ice, icebergs, and ice shelves, along with cavities beneath ocean-terminating glaciers. Ice cover affects air and ocean temperature by reflecting incoming solar radiation and limiting air–sea heat exchange, while the mass loss of ocean-terminating glaciers is one of the main drivers of global sea level increase (Scambos et al., 2017). Brine rejection during sea-ice formation also increases the density of ocean water, driving the global ocean circulation, which exports carbon dioxide from the atmosphere to ocean depths (Goosse and Fichefet, 1999; DeVries, 2022). Under-ice environments host rich and diverse ecosystems (Martínez-Pérez et al., 2022), from algal blooms and cetaceans seeking refuge to top predators (Lund-Hansen et al., 2024; Druckenmiller et al., 2018).

Accurate predictions of future climate, ocean circulation, sea level increase, and species distributions rely on a detailed understanding of ice cover, properties, and melt, which are poorly constrained, in part, due to complex ice–ocean interactions and limited observations (Scambos et al., 2017; Siegert et al., 2020). Glacier melting often occurs in difficult-to-access cavities, which can be less than 1-m thick, below hundreds of meters of ice, and are tens to hundreds of kilometers from the open ocean, as observed on many fast-retreating glaciers in Antarctica (Milillo et al., 2022; Schmidt et al., 2023). The outflow of relatively cold and fresh meltwater can draw warm ocean water into these cavities, accelerating the melting of ice shelves from below (Holland et al., 2020). Maps of ice cavities, observations of under-ice marine organisms, and measurements of ocean physical and biological properties near ice are all necessary to better understand ice melting and ecosystem dynamics.

**FIGURE 1:**
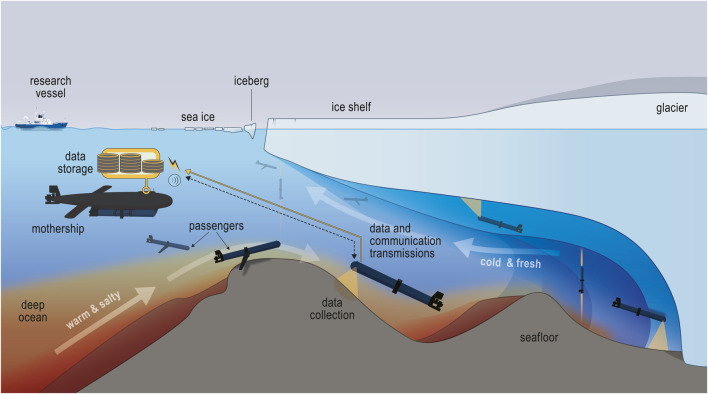
Underwater Mothership–Passenger system exploring an under-ice environment. Multiple Passenger robots deploy from a single Mothership to explore and collect data, forming an *ad hoc* communication network.

Accessing under-ice environments requires highly specialized underwater vehicles such as the Autosub (McPhail et al., 2019) and the Icefin (Meister et al., 2020). With very limited or no surface access, however, these GPS-denied environments present additional challenges to navigation and a greater risk of vehicle loss than in typical ocean environments (Cowing, 2024). Solutions for navigation have included acoustic beacons embedded beneath the ice to guide gliders (Rainville et al., 2020) and floats programmed to exploit opposite flows near the seabed and the ice surface to explore farther into ice shelves and return (Girton et al., 2019). Due to the high costs of these vehicles and floats and the risk of loss, only a few are typically deployed at a time. Here, we explore the scenario in which a more capable underwater vehicle (the Mothership) is deployed in open water and navigates only as far under the ice as necessary—and deemed reasonably safe—to deploy many small, lower-cost vehicles (the Passengers) while also providing multi-agent coordination assistance for this system. Using many lower-cost vehicles would offer greater spatial coverage and de-risk ocean missions by reducing both the cost and data impact of losing any individual vehicle. Figure 1 illustrates this mission concept.

Coordinating the actions of the Passenger robots in this system would typically be accomplished using a centralized or decentralized topology (Wang et al., 2022). In a centralized approach, the Mothership would act as a central planning node to produce joint trajectories for all Passengers. Centralized methods are effective in creating globally coordinated plans but limit the autonomy of the deployed robots, which may be necessary for quickly adapting to environmental dynamics. Alternatively, a decentralized strategy would see each Passenger develop its own plan independently, coordinating with the team through multiple rounds of planning and communication. Although this method maintains each Passenger’s autonomy, global coordination can be hindered if information is slow to propagate throughout the team—a common limitation in underwater domains due to low-bandwidth, high-latency acoustic links (Jakuba et al., 2018).

Existing work has demonstrated underwater coordination strategies through hybrid approaches; however, these methods tend to take on a disconnected hierarchical form, where system coordination occurs only at the Mothership level through mission-level planning or re-tasking (Xu et al., 2025; Pinto et al., 2013). As a result, the influence of the Mothership is limited to coarse updates, while the decentralized planners running on board passenger robots continue to operate largely in isolation. This separation restricts the system’s ability to leverage the Mothership’s global perspective to directly shape Passenger planning decisions. Addressing these limitations requires methods that unify hierarchical coordination with decentralized planning, enabling the Mothership to inject globally informed guidance into Passenger autonomy to solve stochastic, resource-constrained multi-robot decision problems.

In this study, we address this gap by introducing two algorithms that together enable tightly coupled hybrid coordination for underwater Mothership–Passenger teams. The first algorithm targets underwater Passenger robot coordination by incorporating decentralized and centralized solutions to a multi-robot orienteering problem, and it previously appeared in preliminary form as a conference paper at ICRA 2025 (Butler and Hollinger, 2025). This method is included here to establish the hybrid coordination framework and problem setting that motivate and inform our second contribution. Building on this foundation, the second contribution introduces a learned policy on the Mothership that adaptively configures individual Passengers’ priorities, enabling globally informed coordination while substantially reducing inter-robot message passing and supporting more complex mission structures. We evaluate both algorithms in simulated underwater mission environments and conduct hardware deployments using our second algorithm at Fern Ridge Lake in Veneta, Oregon. Our results show that incorporating Mothership guidance into distributed team planning provides the benefits of both centralized and decentralized coordination, achieving heightened team coherence while preserving individual adaptability in complex, dynamic mission scenarios. Although the coordination challenges we address—intermittent communication, stochastic environments, and complex task scenarios—are directly motivated by under-ice deployments, the methods developed here are general and applicable to any multi-robot setting with similar constraints. Under-ice deployment itself involves additional environmental challenges beyond the scope of the current experimental validation, which we discuss explicitly in Section 5. These advances move us closer to the long-term, robust team coordination required for sustained under-ice field deployments.

## 2 Related work

In this section, we review prior work relevant to hybrid underwater multi-robot coordination in Mothership–Passenger systems. We first examine planning and optimization foundations for coordinating underwater robot teams under uncertainty, with particular focus on orienteering-based formulations that capture deployment and return constraints, which motivate our first contribution. We then review role-based and hierarchical coordination methods for multi-robot teams, grounding our second contribution in prior work on adaptive role assignment and learning-based coordination.

### 2.1 Hybrid planning and stochastic orienteering for underwater multi-robot coordination

Foundational multi-robot task allocation (MRTA) taxonomies formalize when coordination is required and how task structure influences planning complexity. Gerkey and Matarić (2004) introduced classical MRTA axes describing robot capabilities and task requirements, while the iTax taxonomy proposed by Korsah et al. (2013) extended this view to explicitly capture task coupling and interdependencies that necessitate coordination. These frameworks underpin much of the work on underwater multi-robot planning, where strong coupling commonly arises from shared energy budgets, communication constraints, and mission-level return requirements.

Centralized planning approaches for autonomous underwater vehicles (AUVs) often jointly optimize task allocation and routing while explicitly modeling constraints such as energy usage, ocean currents, and docking or recharging (Wang et al., 2022; Singh and Hollinger, 2024). These methods enable globally coherent plans but depend on reliable communication and global state estimates, which are difficult to maintain in acoustic environments. In contrast, decentralized approaches—including consensus-based bundle algorithms (CBBAs) and local replanning variants—prioritize robustness to communication delays and failures (Buckman et al., 2019; Chen et al., 2022; Li et al., 2023). Although these methods are effective under intermittent connectivity, they often struggle to enforce globally optimal coordination when information propagates slowly.

Hybrid frameworks seek to balance these tradeoffs by combining centralized planning with decentralized execution. Representative examples include base-station tour generation followed by distributed replanning (MahmoudZadeh and Yazdani, 2023), intermittent rendezvous exploration strategies (Da Silva et al., 2024), and surface-vehicle relays that enable occasional centralized supervision (Jakuba et al., 2018). These approaches align well with underwater operational realities, but they typically rely on deterministic planning assumptions or coarse, episodic coordination updates.

A closely related class of problems arises from the requirement that Passenger robots deploy from and ultimately return to a Mothership. This behavior is naturally captured by the orienteering problem (OP), which requires selecting and visiting a subset of tasks under a resource budget (Gunawan et al., 2016). Extensions such as the stochastic orienteering problem and the stochastic team orienteering problem (STOP) introduce uncertainty in costs and rewards and have been addressed using simheuristics, genetic algorithms, and Monte Carlo simulation (MCS) (Panadero et al., 2020; 2024; Juan et al., 2021; Karunakaran et al., 2019). In distributed settings, deterministic multi-agent OP (MOP) variants have been solved using decentralized Monte Carlo tree search (Dec-MCTS), enabling anytime coordination under lossy communication (Best et al., 2020; Skrynnik et al., 2024).

However, prior MOP formulations do not incorporate stochastic costs or rewards, despite the prevalence of uncertainty in underwater environments due to ocean currents and other environmental dynamics. As a result, existing hybrid and decentralized coordination methods lack a principled mechanism for coupling global guidance with decentralized execution under uncertainty. This gap motivates our first contribution: a hybrid coordination framework that integrates stochastic multi-agent orienteering with decentralized planning for underwater Mothership–Passenger systems.

### 2.2 Role-based and hierarchical coordination for underwater multi-robot teams

Many underwater missions involve coupled objectives that are naturally decomposed into complementary roles, such as exploration, sensing, and communication relay. Role-based coordination frameworks exploit this structure by assigning robots role-specific objectives, whose collective execution maximizes team performance (de Hoog et al., 2009; Scherer and Rinner, 2020). In marine domains, explorer–relay decompositions are commonly used to balance information gain with communication maintenance and data return (Indiveri, 2018; Petillot et al., 2019).

A prevalent approach to role specification is utility scalarization, where heterogeneous objectives are combined via weighted sums or adaptive scalarization strategies (Wilde et al., 2024). In underwater and maritime settings, such methods have been applied to coordinate AUV sensing with ASV relay placement, with weights adjusted in response to link quality or mission priorities (Ren et al., 2023; Mao et al., 2025). Although flexible, scalarization alone does not address how role assignments should be globally coordinated or adapted over time.

Beyond scalarization, role alignment frameworks explicitly coordinate which robots should assume which roles and where they should operate. Examples include connectivity-aware role assignment for relay placement (Abu-Aisheh et al., 2022) and Mothership–Passenger surveillance architectures that partition sensing and coordination responsibilities (Petillot et al., 2019). These methods demonstrate the value of hierarchical structure but typically rely on hand-designed coordination logic or static role definitions.

A growing body of recent work applies large language models (LLMs) to multi-robot task planning, including task decomposition, coalition formation, and high-level plan generation (Chen et al., 2024; Kannan et al., 2024). Although these methods have demonstrated compelling results in settings with rich semantic structure, direct comparison in our evaluation setting is not straightforward for several reasons. First, the planning problems addressed in this work are defined over continuous, stochastic environments with spatially distributed numerical objectives (task richness, communication quality, and exploration coverage). LLM-based planners have primarily been demonstrated in settings with discrete task semantics and natural-language-expressible goals, where their broad world knowledge provides a genuine advantage. Second, the isolated underwater operational environment mandates low-bandwidth observation packets, while current LLM inference imposes latency and compute requirements that are incompatible with onboard or low-power Mothership platforms. We acknowledge that LLM-based planners represent a compelling direction for future work in high-level mission scheduling, particularly as model distillation and edge deployment mature, but a controlled and fair comparison in the current setting is not feasible and is left for future work.

Learning-based approaches offer a mechanism for adapting roles in response to the environment and team state. Centralized training with decentralized execution (CTDE) enables a centralized learner to exploit global information while producing decentralized policies (Yang et al., 2018; Rashid et al., 2018). Role-specialized learning frameworks and transformer-based multi-agent architectures further demonstrate how agent behaviors can be dynamically contextualized within a team (Wang et al., 2020a; b; Wen et al., 2022). However, these approaches have had limited application in underwater domains and rarely consider explicit Mothership–Passenger hierarchies.

Consequently, existing work lacks a unified framework in which a Mothership can learn to adaptively shape decentralized robot behavior by configuring role-specific priorities in real time. This gap motivates our second contribution: a hierarchical learning approach in which a Mothership policy injects globally informed role guidance directly into decentralized Passenger planning.

## 3 Methods

Underwater Mothership–Passenger teams must coordinate Passenger actions for varied levels of task complexity and communication bandwidth. To this end, we present two methods: 1) Mothership-enhanced Passenger orienteering (MEPO), a multi-robot coordination algorithm to solve a multi-agent routing problem tailored for marine exploration, and 2) Mothership-coordinated adaptive Passenger specializations (MCAPSs), a learning-based hybrid-decentralized coordination approach that adapts Passenger behaviors to address more complex mission requirements.

### 3.1 MEPO: Mothership-enhanced Passenger orienteering

The MOP is a highly applicable formulation for coordinating teams of robots for task allocation and path planning problems (Gunawan et al., 2016). MOPs focus on planning robots’ paths between tasks distributed throughout an environment. The objective is to generate an optimal set of schedules by allocating tasks to individual robots and planning each robot’s tour through its assigned task subset such that global task reward is maximized and budget constraints (usually time or energy) are satisfied. In the underwater robotics domain, the MOP is made more difficult by stochastic factors such as communication packet loss, unpredictable travel costs, and random equipment failure (Cai et al., 2023).

To address this, we formulate the stochastic multi-agent orienteering problem (SMOP) (Section 3.1.1) and propose a hybrid solution that combines centralized and distributed planning (Section 3.1.2). In our method, MEPO, the Mothership uses its computational power to develop centralized solutions, while each Passenger generates local plans using lightweight solvers. Passengers can request updated tours from the Mothership to compare against their local plans, sharing the best options across the network for dynamic rescheduling. This approach combines the high-quality solutions of centralized control with the resilience of distributed systems.

#### 3.1.1 Stochastic multi-agent orienteering problem formulation

We consider a team of robots that must coordinate in an uncertain environment to complete spatially distributed tasks. Each task can only be completed once, so revisiting a completed task offers no reward. The team must coordinate to create schedules, or tours, for each member to follow multiple tasks. The energy expended by a robot moving between two locations is difficult to predict but can be modeled using a probability distribution. The objective is to select a set of schedules that maximize the reward gained from completed tasks. However, unpredictable disruptive events may occur during execution, motivating online rescheduling.

##### 3.1.1.1 Problem features and graph representation

We begin formalizing the SMOP by defining a team of M robots $a=\left\{a_{1},a_{2},\ldots a_{M}\right\}$ , with each robot $a_{i}$ assigned an energy budget $b_{i}$ , a start task, and an end task. The environment contains N spatially distributed tasks $v=\left\{v_{1},v_{2},\ldots v_{N}\right\}$ . Each task $v_{j}$ is defined with a deterministic location, work cost $c_{v}^{j}$ , and reward $r_{j}$ , such that when robot $a_{i}$ completes task $v^{j}$ , it reduces its budget by $c_{v}^{j}$ and collects reward $r_{j}$ . The stochastic cost incurred by a robot traveling from task $v_{j}$ to task $v_{k}$ is defined by $c_{e}^{j,k}>0$ , which follows a Gaussian distribution with mean $E\left[c_{e}^{j,k}\right]>0$ . Although ocean current-induced energy variability may be asymmetric or multimodal in complex flow fields, a Gaussian model provides a tractable first-order approximation that captures the mean and variance of energy expenditure, which is consistent with prior stochastic orienteering formulations (Panadero et al., 2020) and enables efficient uncertainty propagation in the Monte Carlo simulation stage of our method.

To represent the problem, we model the environment as a complete graph G={v,e} , where the set of tasks v forms the nodes and the edges e represent the travel costs between tasks. The travel costs are probabilistic and represented by distributions. We assume that the distribution of cost realizations is well represented by a set of scenarios, Ω , where each scenario ω∈Ω contains a set of sampled edge costs, denoted by $\omega =\left\{c_{e}^{0,1},c_{e}^{0,2},\ldots \right\}$ .

##### 3.1.1.2 Scheduling decisions

We solve for a tour, or sequential ordering of nodes, for each robot to follow. A tour for $a_{i}$ is denoted as $\tau_{i}=\left(v_{i}^{1},v_{i}^{2},\ldots \right)$ , and the reduced set of tasks that are uniquely visited by $a_{i}$ is denoted as $u_{i}=\left\{v_{i}^{1},v_{i}^{2},\ldots \right\}$ . With the goal of solving unique tours for each robot, we consider the set of tours $T=\left\{\tau_{1},\tau_{2},\ldots ,\tau_{M}\right\}$ as a solution to the problem in solution space T .

##### 3.1.1.3 Problem statement

The goal is to maximize the total reward accumulated by all robots, considering the stochastic nature of travel costs. For each scenario s , the reward depends on the number of unique tasks completed by each robot, provided that their energy budgets are not exceeded. With this, we determine the optimal set of tours T* that maximizes task completion efficiency. The objective can be expressed in Equation 1:

$$T^{*}=\underset{T\in T}{arg\;max}\frac{1}{\left|\Omega \right|}\underset{\omega \in \Omega }{\sum }\underset{i\in N}{\sum }f\left(\tau_{i},u_{i},\omega \right),$$ (1)

where $f\left(\tau_{i},u_{i},s\right)$ represents the total reward gained from $u_{i}$ , derived as Equation 2:

$$f\left(\tau_{i},u_{i},\omega \right)=l^{\omega }\left(\tau_{i}\right)\cdot \underset{v_{i}^{j}\in u_{i}}{\sum }r_{j}.$$ (2)

The indicator function $l^{s}\left(\tau_{i},s\right)$ enforces budget constraints by forcing the tour’s reward to 0 if the sampled tour cost exceeds $b_{i}$ (Equation 3):

$$l^{\omega }\left(\tau_{i}\right)=\left\{\begin{array}{cc} 1, & \text{ if }b_{i}-\underset{v_{i}^{j}\in \tau_{i}}{\sum }\left(c_{e}^{j-1,j}+c_{v}^{j}\right)>0\\ 0 & \text{ otherwise } \end{array}\right\}.$$ (3)

In the multi-robot setting, each robot seeks the tour ${\tau *}_{i}$ that maximizes its individual contribution to the global reward. We consider the solution space of individual tours $T_{i}$ , such that $\tau_{i}\in T_{i}$ . The utility of robot i ’s schedule is calculated as the difference in the reward with and without i ’s contribution. With this, we optimize Equation 4;

$$\tau_{i}^{*}=\underset{\tau_{i}\in T_{i}}{arg\;max}\frac{1}{\left|\Omega \right|}\underset{\omega \in \Omega }{\sum }g\left(\tau_{i},u_{i},\omega \right),$$ (4)

where (Equation 5)

$$g\left(\tau_{i},u_{i},\omega \right)=\underset{a_{i}\in a}{\sum }f\left(\tau_{i},u_{i},\omega \right)-\underset{a_{j}\in a\backslash a_{i}}{\sum }f\left(\tau_{j},u_{j},\omega \right).$$ (5)

#### 3.1.2 Hybrid mission planning with MEPO

**FIGURE 2:**
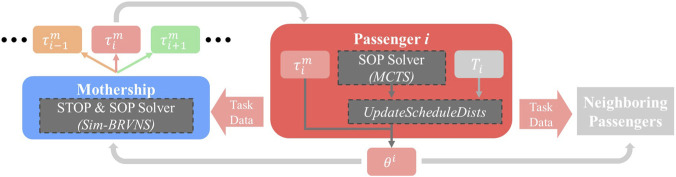
MEPO hybrid-decentralized mission planning routine. The Mothership’s centralized solver generates set $T^{m}$ of tours and sends $\tau_{i}^{m}$ to Passenger $a_{i}$ . On $a_{i}$ , a MCTS solver produces set $T_{i}^{l}$ of tours with local information. New schedules on $a_{i}$ are compared to stored set $T_{i}$ schedules, with the highest-scoring subset of k schedules assigned as new $\left(T_{i},p_{i}\right)$ .

With MEPO, the Mothership uses a centralized solver with its high computational capabilities to enhance a decentralized Passenger robot planning algorithm. Before deploying the Passengers, the Mothership warm-starts its decentralized coordination routines with a centrally solved set of team schedules. Once the mission has started, the Mothership injects individual schedules into the system that are solved using its aggregated global data. MEPO’s key contribution is the UpdateScheduleDist procedure (Algorithm 3), which allows Mothership-generated and locally generated tours to compete directly within each Passenger’s schedule distribution so that the highest-quality plans are continuously selected, regardless of their source. The resulting distribution representation also enables Passengers to communicate probabilistic planning intent to neighbors and the Mothership, supporting coordinated task avoidance without explicit plan negotiation. This section details the algorithms used for scheduling on the Mothership and each Passenger and how planning information is shared across the hybrid system. Figure 2 provides an overview of our hybrid MEPO framework, which guides the following sections.

This framework governs the exchange of scheduling information between all robots in the MRS. The Mothership m runs a centralized solver to generate tours for one or more robots, sharing each tour $\tau_{i}^{m}$ with the respective robot $a_{i}$ . Passenger $a_{i}$ solves a set of k tour options $T_{i}^{l}=\left\{\tau_{i,1}^{l},\tau_{i,2}^{l},\ldots ,\tau_{i,k}^{l}\right\}$ , which it compares against its stored set of tour options $T_{i}=\left\{\tau_{i,1},\tau_{i,2},\ldots ,\tau_{i,k}\right\}$ and the received Mothership tour $\tau_{i}^{m}$ . The local schedule selection algorithm UpdateScheduleDists reduces these to a set of the k best tours and updates $a_{i}$ ’s stored set $T_{i}$ .

Each tour $\tau_{i,j}\in T_{i}$ is associated with a probability $p_{i,j}$ , forming a distribution $p_{i}=\left\{p_{i,1},p_{i,2},\ldots ,p_{i,k}\right\}$ representing the likelihood that $a_{i}$ will follow each tour in $T_{i}$ . When $a_{i}$ ’s tour distribution $\theta_{i}=\left(T_{i},p_{i}\right)$ is shared with other robots, it informs their sampling-based planners about $a_{i}$ ’s likely task visits. Each robot stores a set of received schedule distributions $\theta_{i}=\left\{\theta_{1},\theta_{2},\ldots ,\theta_{N}\right\}$ for reference during planning.

##### 3.1.2.1 MEPO centralized solver

We adopt the Sim-BRVNS algorithm from Panadero et al. (2020) to solve the STOP on the Mothership.

The SimBRVNS function solves the STOP for a team of M robots using the following steps:

1. Converting the STOP into a deterministic problem using the mean values of edge cost distributions.
2. Solving this deterministic problem using a constructive heuristic.
3. Evaluating the solution in a stochastic setting via MCS, where edge costs are sampled from the Mothership’s graph $G_{m}$.
4. Applying a biased-randomized variable neighborhood search to explore additional solutions.

The solver iterates between steps 3 and 4 within a given time limit, returning the best-performing set of M schedules based on the evaluation function (Equation 1). For more details on this method, refer to Juan et al. (2022) and Panadero et al. (2024).

In the SMOP - SimBRVNS function, we extend Sim-BRVNS to the multi-robot SMOP case using the schedule distributions from $\theta_{m}$ stored on the Mothership. In this case, the Mothership solves a single tour for $a_{i}$ . During each iteration of the MCS stage, the solver samples a set of tours for all robots, except $a_{i}$ . The sampled tours and the current solution are evaluated on $G_{m}$ , and the utility reward for $a_{i}$ ’s tour is calculated using Equation 4. At the end of the solver’s runtime, the highest-scoring solutions $T_{i}^{m}$ are returned.

##### 3.1.2.2 MEPO distributed solver

We select the Dec - MCTS algorithm proposed by Best et al. (2020) to solve local tour solutions on each Passenger $a_{i}$ . The algorithm grows a search tree by cycling through four phases: selection, expansion, simulation, and backpropagation while sharing partial solution information between robots. Each node in the tree represents a tour, with the root node corresponding to the Passenger’s current location. The algorithm returns the set of the k best-performing tours $T_{i}^{l}$ . For further information on Dec-MCTS, refer to Best et al. (2020).

We apply this algorithm to the SMOP by adding an additional sampling layer to the rollout phase. During this phase, multiple rollouts of the leaf node’s tour are performed. The rollout process simulates adding valid tasks to the tour, maximizing the ratio of added reward to incurred travel cost. These travel costs are sampled from the local graph $G_{i}$ . Nodes are considered valid only if they are not already included in tours sampled from $\theta_{i}$ at the start of the rollout. The local utility reward of the simulated tour is then calculated against the sampled tours using Equation 4.

##### 3.1.2.3 MEPO hybrid scheduling algorithms

Two distinct algorithms, Algorithm 1 (Mothership rescheduling) and Algorithm 2 (Passenger rescheduling), run on the Mothership and each Passenger, respectively. Algorithm 1 enables the Mothership to solve either the STOP for team scheduling (line 2) or an individual robot’s schedule for online SMOP (line 6). The STOP is used at the mission’s outset to generate an offline mission plan for each robot, while tours for the SMOP are solved during runtime for $a_{i}$ as requested. Basic communication functions, Send(Content,Receiver(s)) and Receive(Content,Sender) , are used to share data between robots.

Algorithm 1 **Centralized Mothership scheduling algorithm for SMOP.**

**Input:** robots a , task graph $G_{m}$ , stored schedule distributions $\theta_{m}$

1:  **if** mission is beginning **then** ⊳ Run on startup
2: $T^{m}\leftarrow SimBRVNS\left(G_{m},a\right)$

3: $Send\left(T^{m},a\right)$ ⊳ Distribute tours
4:  **end if**
5:  **if** $Receive\left(request,a_{i}\right)$ **then** ⊳ Run as requested by $a_{i}$

6: $\tau_{i}^{m}\leftarrow SMOP$ - $SimBRVNS\left(G_{m},a_{i},\theta_{m}\right)$

7: $Send\left(\tau_{i}^{m},a_{i}\right)$ ⊳ Return solved tour
8: **end if**

Algorithm 2 manages rescheduling for each Passenger $a_{i}$ . Upon startup, $a_{i}$ receives an initial tour from the Mothership and begins executing it (lines 1–5). During runtime, $a_{i}$ can request updated schedules from the Mothership (line 6). If a new schedule is received, $a_{i}$ updates its schedule distribution (lines 7–9). If no update is provided, the Passenger runs Dec - MCTS to generate local solutions (line 10). These local options are then evaluated against stored schedules using the UpdateScheduleDist function (line 11), as described in the next section. Finally, the Passenger shares its updated schedule distribution with the Mothership and nearby Passengers (line 14) and then returns the best-performing schedule from the new distribution for execution.

These algorithms equip the system to operate as a centralized network when Passengers maintain a consistent connection to the Mothership by granting centrally generated plans precedence over local tours. Additionally, Algorithm 2 enables Passengers with strong connections to neighboring robots to develop plans as a distributed system by exchanging schedule distributions.

Algorithm 2 **Decentralized Passenger $a_{i}$ scheduling algorithm for SMOP.**

**Input:** robots **r**, task graph $G_{i}$ , stored schedule distributions $\theta_{i}$ , stored local schedules $T_{i}$

**Output:** new schedule $\tau_{i}$

1:  **if** mission beginning **then** ⊳ Receive init. tours
2: $\tau_{i}\leftarrow$ extract $\tau_{i}^{m}$ from $Receive\left(T^{m},m\right)$

3: $\theta_{i}\leftarrow \left(\left[\tau_{i}\right],\left[1.0\right]\right)$ ⊳ Init. distribution $\left(T_{i},p_{i}\right)$

4: $return\;\tau_{i}\leftarrow \underset{\tau_{i}\in T_{i}}{\arg\max}\left[p_{i}\left(\tau_{i}\right)\right]$

5:  **end if**
6: Send(request,m) ⊳ Request tour from M
7:  **if** $Receive\left(\tau_{i}^{m},m\right)$ **then** ⊳ Use received tour
8: $\theta_{i}\leftarrow \left(\left[\tau_{i}^{m}\right],\left[1.0\right]\right)$

9:  **else** ⊳ Else solve local tours
10: $T_{i}^{l}\leftarrow Dec$ - $MCTS\left(G_{i},a_{i}\right)$

11: $\left(T_{i},p_{i}\right)\leftarrow UpdateScheduleDist\left(T_{i}\cup T_{i}^{l}\right)$

12: $\theta_{i}\leftarrow \left(T_{i},p_{i}\right)$

13:  **end if**
14: $Send\left(\theta_{i},a\cup m\right)$ ⊳ Share new schedule distribution
15: $return\;\tau_{i}\leftarrow \underset{\tau_{i}\in T_{i}}{\arg\max}\left[p_{i}\left(\tau_{i}\right)\right]$

##### 3.1.2.4 Updating schedule distributions

The UpdateScheduleDist function evaluates both old and new schedules developed for $a_{i}$ to determine the best subset to carry forward. Algorithm 3 outlines the procedure used to assess and select the top schedules from a set of candidates. The algorithm iterates through each candidate schedule τ , first modifying τ according to locally stored information with Prune (i.e., removing tasks that $a_{i}$ knows have been completed).

A Monte Carlo simulation is then performed using local budget constraints and graph information to assess the failure probability of τ across multiple scenarios (line 4). Next, LocalUtil evaluates the local utility of τ using Equation 4. The utility and failure probability are combined into a score α for each tour (lines 5 and 6). The algorithm stores each tour–score pair in a list (line 7). Tours are then sorted by α and reduced to a list of the top k candidates (line 9). Finally, the scores α are normalized to create a probability distribution $p_{i}$ for the selected schedules $T^{\prime }$ .

Algorithm 3 **UpdateScheduleDist method for integrating new and stored local SMOP solutions.**

**Input:** tours to evaluate T , stored schedule distributions $\theta_{i}$ , budget $b_{i}$ , task graph $G_{i}$ , stored tour count k

**Output:** new schedule distribution $\left(T_{i},p_{i}\right)$

1: pairs←∅

2:  **for** τ∈T **do**
3: $Prune\left(\tau ,G_{i}\right)$ ⊳ Prune completed tasks
4: $rel\leftarrow MCS\left(\tau ,b_{i},G_{i}\right)$ ⊳ Evaluate failure rate
5: $rew\leftarrow LocalUtil\left(\tau ,\theta_{i}\right)$ ⊳ Equation 4
6: α←rew⋅rel ⊳ Compute tour score
7: pairs←pairs∪(τ,α) ⊳ Add to pairs
8:  **end for**
9: $\left(T^{\prime },\alpha \right)\leftarrow SelectTopSchedules\left(pairs,k\right)$

10: α←Normalize(α)

11: $return\;\left(T^{\prime },\alpha \right)$

### 3.2 MCAPS: Mothership-coordinated adaptive passenger specializations

As discussed in the previous section, MEPO focuses on routing a multi-robot team for simple, single-robot tasks, where each task is considered complete once it is visited by any individual robot. Additionally, the decentralized component of the MEPO framework relies on intensive message passing between Passenger robots to exchange up-to-date schedule distributions. Although these constraints are acceptable for some mission scenarios, many real-world deployments may require algorithms that generalize to a wider variety of tasks and with less communication overhead.

These challenges motivate MCAPSs, a flexible method of multi-robot role coordination that learns adaptive, high-performing Passenger robot behaviors without relying on fixed assignments (Section 3.2.1). MCAPS supports adaptive Passenger behaviors using a scalarized multi-objective problem formulation (Section 3.2.2). The Mothership acts as a high-level coordinator using a learned policy that generates Passenger-specific role assignments tailored to the current problem configuration (Section 3.2.3). These assignments are then integrated asynchronously into Passengers’ local planners, allowing for globally informed actions without requiring explicit planning communication between deployed agents. As a result, Passengers need only to connect with the Mothership to periodically upload data and receive updated role assignments, significantly reducing messaging requirements when compared to MEPO.

#### 3.2.1 MCAPS framework overview

**FIGURE 3:**
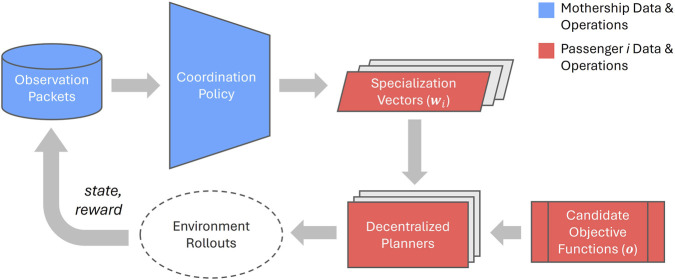
Framework for MCAPSs. Location-tagged regional observation data packets from the environment are aggregated at the Mothership and processed to produce specialization scalarization vectors for each Passenger. Passengers use these to scalarize their local candidate objective functions into a single objective, which they then use in their decentralized planners to interact with the environment.

The goals of MCAPS are to maintain Passengers’ decentralized, local autonomy to respond quickly to local disturbances while allowing the Mothership to influence coordinated team behaviors by manipulating individual Passengers’ local objectives. The full MCAPS framework (Figure 3) encompasses operations at both the Mothership and Passenger levels. The Mothership is responsible for extracting valuable information from the observation packets that it has received from deployed Passenger robots. It uses this information to generate coordination in the form of specialization vectors for each robot in the team, where vectors contain weights corresponding to a set of candidate objective functions provided to each Passenger. Once a Passenger receives and applies its specialization vector, the resulting weighted objective function guides its actions in the environment.

#### 3.2.2 Passenger robot planning with scalarized objectives

Each Passenger robot is equipped with a local planner that guides its decision making throughout the mission. Due to real-world sensing and communication limits, we assume that Passengers cannot rely on continuous access to global information or coordination signals. Instead, they must operate autonomously between updates from the Mothership.

##### 3.2.2.1 Decentralized multi-objective planning problem formulation

To support their autonomy while enabling system-wide coordination, we adopt a planning framework in which each Passenger’s planner is guided by a scalarized objective function. A Passenger’s specialization vector—assigned by the Mothership—defines the robot’s behavior by encouraging it to prioritize different objectives based on current mission needs.

We define a set of domain-inspired candidate objectives that represent distinct environmental or task-related features observable by the Passengers. These objectives may include values such as distances to nearest tasks, proximity to other agents, coverage of unexplored areas, quality of communication links to the Mothership, and more. Passenger i ’s planner combines these objectives into a single-valued function using its specialization vector $w_{i}\in R^{O}$ , where O is the number of objectives. The resulting scalarized objective function allows the planner to flexibly prioritize different behaviors.

Formally, given a locally observed set of candidate states $V_{i}$ , where each $v_{i}\in V_{i}$ represents a state with associated objective values $o\left(v_{i}\right)\in R^{O}$ , a Passenger’s objective is to compute a path $\tau_{i}\subseteq V_{i}$ to goal state ${v*}_{i}$ , defined dynamically using the minimum weighted sum of these values, as shown in Equation 6:

$$v_{i}^{*}=\underset{v_{i}\in V_{i}}{\min}w_{i}^{T}o\left(v_{i}\right).$$ (6)

A Passenger then solves for a minimum-cost path τ* from its current location to ${v*}_{i}$ (where $c\left(v_{i},v_{j}\right)$ provides the cost to transition between states $v_{i}$ and $v_{j}$ ). This formulation enables each Passenger to adapt its behavior to a specific specialization defined by its assigned $w_{i}$ while retaining the ability to plan and replan independently based on its local observations. By modifying these vectors, the Mothership can coordinate complex multi-robot behaviors without requiring centralized trajectory planning or continuous inter-agent communication.

##### 3.2.2.2 Decentralized planning algorithm with adaptive specializations

A Passenger i uses its received specialization vector in its local planner through the following process (Algorithm 4): first, the passenger extracts a set of candidate states from its locally observed environment with GetCandidateStates (line 1). For discrete environment representations, these states may be nodes in a graph. For continuous environments, a fixed number of states may be randomly sampled. Next, the Passenger searches its candidate states for the state that maximizes the weighted sum of its specialization vector parameters and candidate objective values (lines 2–8). With this goal state, the Passenger uses a local planner Plan to find a sequence of actions to reach that state (line 9) and returns the resulting plan (line 10).

Algorithm 4 **Decentralized planning with adaptive specializations.**

**Input:** Local environment $env_{i}$ , specialization vector $w_{i}$ , objective functions o , planner Plan , starting state $v_{s}$ , cost function c

**Output:** Path $\tau_{i}$

1: $V_{i}\leftarrow GetCandidateStates\left(env_{i}\right)$ ⊳ Extract candidate states from env
2: ${v*}_{i}\leftarrow None$ , val*←inf ⊳ Init. null goal, inf value
3:  **for** $v\in V_{i}$ **do**
4: $val\leftarrow w_{i}^{T}o\left(v\right)$ ⊳ Eval. specialization value with 6
5: val<val* **then**
6: ${v*}_{i}\leftarrow v$ , val*←val ⊳ Update goal and best value
7:    **end if**
8:  end **for**
9: ${\tau *}_{i}=Plan\left(env_{i},v_{s},{v*}_{i},c\right)$ ⊳ Create minimum-cost plan to goal state
10: $return\;{\tau *}_{i}$

When a Passenger’s local observations change or it receives new specialization vector, Algorithm 4 enables the robot to update its behavior with a new goal state and local plan.

#### 3.2.3 Learning to coordinate passenger objectives

With decentralized planning behaviors and specialization vectors defined for each Passenger, the remaining challenge is to coordinate these specializations so as to maximize overall team performance. The Mothership’s policy must aggregate sparse, location-tagged observation packets and translate them into globally coherent specialization vectors—a mapping whose optimal form depends on complex, context-dependent interactions among environment dynamics, task structure, and team state that are difficult to capture with hand-designed rules or static optimization. Framing this problem as a reinforcement learning task allows the Mothership to discover coordination strategies that adapt to uncertainty, partial observability, and the delayed effects of specialization decisions by directly optimizing long-horizon team-level rewards through experience. In doing so, MCAPS can discover emergent specialization coordination policies that balance exploration, exploitation, and robustness in dynamic multi-robot mission scenarios.

##### 3.2.3.1 MDP for learning specialization vectors

To learn to coordinate the team, we formulate the Mothership’s decision-making process as a Markov decision process (MDP), in which the Mothership periodically creates behavior specialization vectors for each Passenger. These vectors guide each Passenger’s local planner for a fixed K -step rollout, during which the robots independently create and execute local plans. Our goal is to learn a Mothership policy that selects joint role assignments such that the resulting team behavior maximizes mission performance over an episode comprising T rollouts, measured by the cumulative reward obtained from completing tasks at each step.

We intentionally avoid using dense or heavily shaped reward functions to preserve the opportunity for diverse and emergent strategies to arise. Many real-world missions involve sparse feedback (e.g., rewards may only be issued when a task is completed and its data successfully returned to the Mothership). As such, we design the Mothership MDP to reward only task completions and their corresponding data returns, avoiding intermediate or manually tuned reward signals that could bias the learned policy toward suboptimal behavior patterns.

With this, we define the Mothership MDP as follows:

- **State**: A set of regional observation packets $s_{t}=\left\{s_{t}^{0},s_{t}^{1},\ldots ,s_{t}^{M}\right\}$ provided via periodic updates from each Passenger. Each packet in this representation is tagged with the location with a spatially defined region and includes a vector of regional feature information assembled by the source Passenger (e.g., task richness, traversability, and passenger density).
- Action: A set of specialization parameter vectors $w_{t}=\left\{w_{t}^{0},w_{t}^{1},\ldots ,w_{t}^{M}\right\}$ for each of M Passenger robots.
- **Reward**: Actions occur over an extended time horizon. Thus, our reward function for evaluating the impact of these actions considers a K -step rollout. For each of N tasks, the global reward gained from an individual task $task_{j}$ at step k is computed using a function $task_{j}\left(H_{k}\right)$ that evaluates the team’s joint state at step k , $H_{k}$ , and returns the corresponding reward $\left(r_{t}\right)$ . In our experiments, $r_{t}$ is weighted by the effective data transfer value, which decays exponentially with the total communication chain distance between the visiting Passenger and the Mothership at the time of task completion. Overall, the cumulative reward over a K -step rollout is presented in Equation 7:

$$r_{t}=\overset{t+K}{\underset{k=t}{\sum }}\overset{M}{\underset{j=0}{\sum }}task_{j}\left(H_{k}\right).$$ (7)

The objective of the Mothership’s policy $\pi_{\theta }\left(s_{t}\right)=w_{t}$ is to select specialization vectors that maximize the expected sum of rewards over the mission duration. With this, the optimal policy π* maximizes the expected rewards obtained over a T -step episode, as shown in Equation 8:

$$\pi^{*}=max\;E\left[\overset{T}{\underset{t=0}{\sum }}r_{t}\right].$$ (8)

This hierarchical structure balances centralized coordination and decentralized autonomy. It enables scalable multi-robot planning in communication-limited, dynamic environments while remaining robust to sparse reward signals.

##### 3.2.3.2 Specialization coordination policy

The Mothership’s coordination policy model must combine observations from multiple Passenger robots and extract useful features to provide coordinated specialization vectors. Transformer models provide an efficient means of propagating information between multiple observation tokens (Vaswani et al., 2017). The attention mechanism used in transformer models enables the policy to learn which components of multiple Passenger updates are most relevant to specialization assignments while ignoring unhelpful features. Similarly, the attention mechanism has been shown to support coordinated action assignments between agents in a multi-agent team (Wen et al., 2022). Our method takes advantage of attention with a transformer model through the following procedure:

1. **Encoder**: Each regional observation packet in $s_{t}$ is tagged with its spatial information (e.g., x–y location of its corresponding region) and then embedded into a latent space to generate the model’s tokens. The transformer encoder then performs self-attention across these tokens to exchange environment information between all regions.
2. **Regional token extraction**: With each region’s token having attended to all other regions’ tokens, we then extract the tokens corresponding to each robot’s location in the environment. These become our *robot tokens* and will be used to produce globally informed, robot-specific specializations.
3. **Decoder**: The decoder layer first performs self-attention between the extracted *robot tokens* before attending to the *robot tokens* with all environment region tokens to reinforce global characteristics. The output of this layer is *robot tokens* that have both attended to each other to draw out potential specialization-specific information and been attended to by the global observation space to reinforce each Passenger’s role in the global system.

Finally, the *robot tokens* are passed through a linear layer to produce robot-specific specialization vectors.

## 4 Results and discussion

In the following sections, we evaluate our MEPO and MCAPS methods in simulated environments and discuss a real-world deployment on an inland lake, in which MCAPS was used to coordinate a multi-robot team consisting of a BlueROV and Platypus Lutra Unmanned Surface Vehicle (USV). We first conduct an evaluation of the MEPO algorithm for enhancing decentralized Passenger robot orienteering in an underwater domain (Section 4.1). We then increase the task complexity of our simulation domain and assess the ability of MCAPS to provide coordinated behaviors before demonstrating a real-world MCAPS hardware deployment (Section 4.2).

### 4.1 MEPO experiments

We evaluate our Dec-MCTS SMOP solution and two versions of our hybrid Mothership–Passenger algorithms, alongside a centralized, offline STOP solver, defined as follows:

- Sim-BRVNS (fully centralized): Solve STOP with Sim-BRVNS to generate a full team schedule offline prior to mission execution. No online optimization (Panadero et al., 2020).
- Dec-MCTS (fully decentralized): Use Dec-MCTS to solve local schedules without input from Mothership. No initial offline scheduling and no online hybrid rescheduling (Best et al., 2020).
- 2-Stage (Ours): Solve initial offline schedules and then perform local-only rescheduling during deployment.
- MEPO (Ours): Solve initial offline schedules and then reschedule using local solutions and requested solutions from Mothership (Algorithm 1 and Algorithm 2).

#### 4.1.1 MEPO evaluation methods

We motivate our experiments within the context of an under-ice environment. The operational area is modeled as a 25 km × 35 km 2D grid, sampled from simulated under-ice ocean current data generated by Si et al. (2024). Each test environment is randomly drawn from a pool of 20 datasets. Ocean currents are unknown to the robots at the start of the mission and influence vehicle movements by increasing energy consumption when traveling against strong flows. Robots traveling with the current experience less energy drain. All robots are assumed to move at a constant velocity. Robot energy budgets calibrated to support tours of approximately 60%–70% of the maximum possible environment traversal. Given uncertain energy cost predictions due to unknown currents, robots model movement costs as stochastic.

Communication between robots occurs at a fixed frequency, with message success rates impacted by underwater absorption and signal spreading. We approximate packet loss as an exponential decay function of the distance between transmitting and receiving robots, with packet success probabilities decreasing to 80% at 5,000 m (Kuperman and Roux, 2007). Beyond this range, the success rate rapidly decreases to the point where messaging attempts are no longer viable.

**FIGURE 4:**
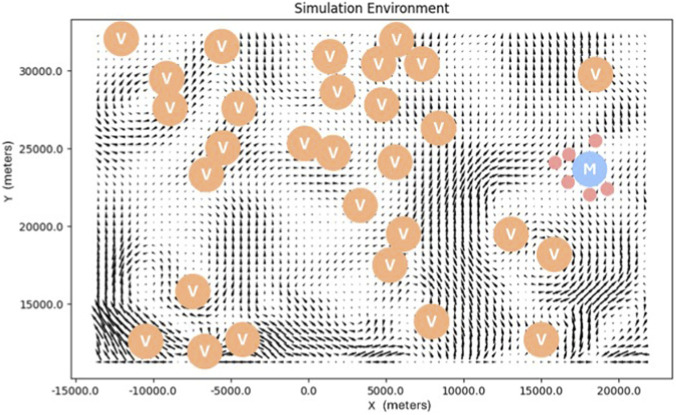
Example simulation environment with sampled flow data, random task locations (Vs), and random Mothership location (M) with deployed Passenger robots (red).

The simulated MRS consists of a Mothership, Worker robots, and Support robots. Each Worker is provided with a team of Support robots, who provide infrastructure for communication and localization by distributing themselves evenly between the Mothership and the Worker. The Mothership and Workers focus on coordinating tours to randomly distributed tasks. The Mothership serves as both the starting and ending point for each Worker’s schedule, and its position is randomly assigned at the start of each test. Figure 4 shows an example simulation environment, including the randomized Mothership location (M) and task locations (Vs). During operation, Workers share completed task information with the Mothership and neighboring robots. In the online rescheduling cases, a Worker communicates a tour update each time it completes a task, or otherwise at a fixed rate of once per 25 steps during runtime, with packet success determined by the exponential decay function described above.

For reproducibility, we provide the following implementation details for the MEPO evaluation. The Monte Carlo evaluation in Sim-BRVNS uses 100 scenarios per solution evaluation, and the solver is given a time budget of 30 s per call for the offline STOP solve and 10 s per call for online SMOP rescheduling. Dec-MCTS and UpdateScheduleDist each execute 50 Monte Carlo evaluations over 10-s run times.

We evaluate each algorithm’s ability to coordinate teams of robots in this stochastic environment and strain each method’s replanning routines by increasing levels of disruption with the following tests.

1. Robot failures: Initialize 30 tasks. At each simulation step, there is a probability (0%, 2.5%, or 5%) that a random worker will experience a failure, resulting in zero, some, or most robots being inactive by the end of runtime, respectively. This failure has a 50% chance of either fully immobilizing the worker or partially reducing its remaining battery life by 50%.
2. New tasks: Initialize 20 tasks. At each simulation step, there is a probability (0.0%, 5.0%, or 10.0%) that the Mothership would generate a new task, resulting in the problem size increasing by approximately 0%, 50%, or 100% throughout runtime, respectively.

Finally, we assess the algorithms’ performance in each test with teams of three and six Workers to observe scalability trends between group sizes. All algorithms are evaluated under identical scenario seeds and given identical compute budgets on the same hardware.

#### 4.1.2 Hybrid-decentralized multi-robot orienteering results

**FIGURE 5:**
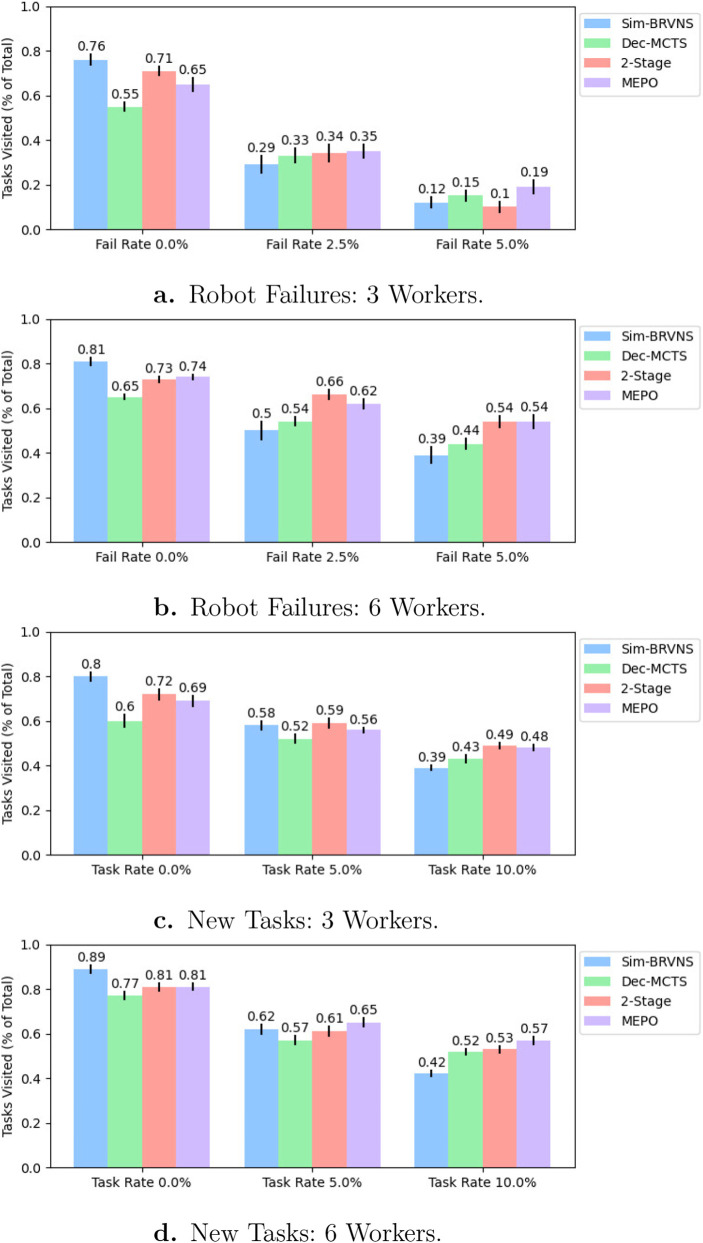
**(a,b)** Algorithm comparison results for teams of three and six Worker robots operating in a 30-task environment with increasing per-timestep robot failure probability. **(c,d)** Algorithm comparison results for teams of three or six Worker robots operating in a 20-task environment with increasing per-timestep new task introduction probability. Results show mean task completion efficiency with a standard error over 30 trials. As environment dynamics increase, the utility of the initial offline Sim-BRVNS solution degrades, while hybrid approaches remain robust.

For each test case, the ACH algorithm was evaluated across 30 independently sampled random environments using a fixed set of random seeds, with all algorithms evaluated on the same 30 environment instances. We report the mean and standard error of the mean across these trials. Sim-BRVNS results are deterministic given fixed scenario seeds; variability in Dec-MCTS and MEPO results reflects randomness in the MCTS rollout process. Figure 5 presents the task efficiency rate results, or the mean task rewards gained by all Workers that successfully completed tours through the environment and returned to the Mothership, according to Equation 1.

We observe the following high-level trends in the test results. First, in the baseline scenarios (0.0% robot failures or new tasks), the offline Sim-BRVNS solver achieves the highest mean reward, consistent with the results expected from the powerful STOP solver. Our 2-Stage and MEPO algorithms follow, while Dec-MCTS performs the worst in every baseline test, highlighting the benefit of an initial planning stage. Next, as disturbances are introduced into the environment via failures and new tasks, the efficacy of the initial offline plan tends to decrease at a higher rate than that of the other methods. On average, the reward obtained by Sim-BRVNS decreased by 60.0% between the baseline and high-disturbance scenarios across all tests. The 2-Stage method experienced the next highest average decrease of 44.6%, followed by MEPO at 39.5% and Dec-MCTS at 32.4%. These results underscore the robustness provided by replanning in highly dynamic scenarios. Finally, in the high-disturbance cases (5.0% robot failures or 10.0% new tasks), we observe that MEPO completes tasks most efficiently. In particular, MEPO achieves a 26.6% mean reward improvement over Dec-MCTS in the three-Worker 5.0% failure test and a 7.5% increase over 2-Stage in the six-Worker 10.0% task rate test, while performing competitively with 2-Stage in the other high-disturbance scenarios. These results suggest that the hybrid rescheduling phase is effective in bolstering the quality of rescheduled solutions in highly dynamic scenarios.

The results also draw out differences between the offline STOP solution and our online SMOP approaches. In all baseline scenarios, performance decreases from Sim-BRVNS to our 2-Stage rescheduling methods. Despite starting with a highly optimal offline plan, the 2-Stage approaches allow Workers to replan conservatively by selecting low-risk tours that skip high-risk tasks. Additionally, 2-Stage outperforms MEPO in many low-to-medium disruption scenarios. This is likely the result of two aspects: 2-Stage following the initial offline solution longer than MEPO due to low-quality Dec-MCTS solutions being rejected by Algorithm 2, and MEPO lacking sufficient information to produce Sim-BRVNS plans that improve on the initial solution due to communication limits. As a result, MEPO performs best when environmental dynamics diminish the utility of the initial plan and when the mothership is well informed.

The communication load of MEPO differs structurally from that of Dec-MCTS. Dec-MCTS performs optimally when all Passengers exchange full schedule distributions at every planning step, scaling with both team size and planning frequency. MEPO and 2-Stage reduce peer-to-peer messaging: in the hybrid rescheduling phase, Mothership-generated tours substitute for multiple rounds of plan negotiation, although they increase the Mothership’s own communication load in high-disruption scenarios where rescheduling is frequent. The results also surface a sensitivity to Mothership information freshness: MEPO’s advantage over 2-Stage is largest precisely under high-disruption conditions, where robot failures and new task arrivals are communicated to the Mothership as they occur, keeping its global picture current (Figures 5a–d). Both the communication-load tradeoff and this information-quality relationship warrant direct quantification in future experiments, particularly in bandwidth-limited acoustic environments.

Overall, our results show that the 2-Stage and MEPO algorithms consistently perform best in the presence of disruptive events. Both approaches scale well across different problem sizes as their distributed nature allows computation to be performed in parallel across the Mothership and all Passengers. However, we anticipate diminishing performance with larger instances as anytime solvers may struggle to find high-quality solutions within limited solving time. Initial offline planning improves performance in most scenarios, and online replanning maintains this advantage under dynamic conditions. In highly dynamic cases, MEPO’s distributed scheduling approach, which integrates centralized and local planning, achieves the best results.

### 4.2 MCAPS experiments

We evaluate our MCAPS method in simulated experiments inspired by autonomous marine exploration and deploy our algorithm on a multi-robot team consisting of a Platypus Lutra USV and Blue Robotics BlueROV. We begin this section by describing the simulation environment, Passenger specializations, and Mothership policy training architecture. We then evaluate how architectural choices for the Mothership’s coordination policy affect performance, how well policies adapt to varying problem configurations compared to fixed-behavior counterparts, and how simulation-trained policies transfer to hardware in real-world lake deployments.

#### 4.2.1 MCAPS simulation environment

Our MCAPS simulation environment is motivated by multi-robot exploration and complex task execution deployments. Specifically, we consider situations in which robots first quickly canvas multiple regions to gather information about environmental characteristics before being sent to specific locations for data collection tasks, such as benthic habitat mapping (Shields et al., 2020; Modasshir et al., 2018), hydrothermal vent exploration (Okamoto et al., 2019), or under-ice surveys in polar environments (Fan et al., 2023).

In our simulation, the objective is for Passenger robots to transmit data collected at task sites back to the Mothership. Due to the limitations of underwater acoustic communication, however, Passengers are required to reduce their messaging bandwidth as their distance from the Mothership increases. We model this effect by assigning a full value (1.0) to data transmitted within close range of the Mothership, with the effective data value decreasing exponentially with distance and reaching 0.0 beyond a defined close-range communication threshold. This limitation can be mitigated when one or more additional Passengers position themselves between the transmitting robot and the Mothership. We assume that these intermediate Passengers can relay messages, thereby increasing the effective data transfer volume and enabling information from distant task sites to be returned to the Mothership.

Formally, our MCAPS simulation environment is built on the vectorized multi-agent simulator (VMAS) to support efficient reinforcement learning (RL) training (Bettini et al., 2022). The environment consists of a grid of regions that Passengers can explore. These regions can contain environment features observed both locally (obstacles and frontiers) and globally (tasks and robots). When a region is visited by a Passenger, that Passenger sends an observation packet containing the region’s location and a summary of its characteristics to the Mothership, which stores the observation alongside packets received from other Passengers. Explored regions may spawn tasks, providing a reward when visited by Passengers. The value of this reward decays to zero according to the maximum communication message distance evaluated in the chain of robots between the visiting Passenger and the Mothership. The Mothership’s location is initialized randomly in the environment, and Passengers deploy from this position.

Finally, to facilitate unique behaviors, Passengers are provided candidate objective functions that evaluate the Euclidean distance from a given location to the following:

1. Nearest task
2. Nearest robot
3. Nearest frontier
4. Nearest communication point (midpoint between two robots)
5. Neediest communication point (midpoint between the Mothership and the farthest robot from the Mothership)
6. **Mothership**

Once a Passenger’s goal location is identified using these functions and Equation 6, it uses a local sampling-based RRT planner to efficiently navigate to the goal in a continuous environment (LaValle, 1998).

#### 4.2.2 Coordination policy training configuration

We optimize the Mothership’s coordination policy using proximal policy optimization (PPO) (Schulman et al., 2017). Three properties of the problem setting motivate this choice. First, because Passenger updates arrive asynchronously and incompletely, the Mothership acts under partial observability, and PPO’s clipped gradient updates provide stable training under the resulting non-stationarity. Second, specialization decisions influence multiple Passengers over several planning steps before any reward signal is observed; PPO’s generalized advantage estimation (GAE) (Schulman et al., 2015) smooths these delayed returns, reducing variance without excessive bias. Third, each training rollout requires simulating all Passengers jointly, so PPO’s ability to perform multiple gradient epochs per collected batch reduces the number of costly environment interactions required.

In our hierarchical Mothership–Passenger framework, the actor network corresponds to the policy described in the previous section, while the critic network is implemented as a second transformer-based model that receives the same Passenger observation packets as input and predicts the expected long-term return. This architecture allows the critic to leverage spatial and relational features of the environment. Both actor and critic networks share similar embedding and attention layers, although the critic omits the per-Passenger decoding step used by the actor. At inference time, specialization vectors for all Passengers are produced in a single forward pass and recomputed once per-Passenger reporting interval, imposing negligible latency relative to the K -step planning rollout.

Table 1 summarizes the key training parameters used in our PPO configuration.

**TABLE 1:** Key PPO training parameters for the coordination policy.

Parameter	Value/Description
Discount factor	0.99
GAE parameter	0.95
Clip ratio	0.2
Entropy coefficient	0.01
Batch size	256
Frames per batch	2,048
Total frames	250,000
Number of epochs per update	16
Optimizer	Adam, learning rate $1\times 10^{-4}$

Additional architectural details for reproducibility are as follows. Each regional observation packet contains six-dimensional feature vectors encoding task richness, obstacle density, Passenger count, and frontier count for the corresponding region, concatenated with a 2D spatial embedding of the region’s grid coordinates. The transformer encoder and decoder each use two layers, with four attention heads and a 128-dimensional latent space. Each episode consists of T=16 Mothership decision steps, with each Passenger executing a K=128 -step planning rollout between Mothership updates. Rollout rewards are not clipped.

#### 4.2.3 Model ablations

To isolate the contribution of key model components, we perform ablations that remove or modify aspects of the transformer architecture and specialization mechanism. As a baseline, we evaluate a feed-forward neural network (linear) that operates only on the observation packets sent from individual Passengers, without allowing information to be exchanged. Next, we evaluate a coordination model that uses only the encoder portion of the transformer, which allows Passengers’ observation packets to attend to each other. To examine the impacts of cross attention between robot tokens alongside global observation attention, we include the full encoder–decoder transformer model. Finally, we evaluate a specialization post-processing method that assigns the maximum value of the specialization vector to 1.0 and all other values to 0.0 for each Passenger to enforce single-role behaviors (Max Action).

Each method was trained using identical parameters and evaluated on 30 identical randomly generated environments. We report the mean and standard deviation of the completed tasks returned to the Mothership for each model configuration in Table 2.

**TABLE 2:** Coordination policy ablation study. Values report mean ( ± standard deviation) of task data returned to Mothership over 30 independently evaluated environments with fixed random seeds. Higher is better.

Linear	Encoder	Decoder	Max action	Task data to mothership
✓	✗	✗	✗	22.0 ( ± 5.6)
✓	✓	✗	✗	20.9 ( ± 5.8)
✓	✓	✓	✗	27.6 ( ± 6.3)
✓	✓	✓	✓	26.4 ( ± 6.7)

The ablation results highlight the importance of global information exchange and specialization flexibility in achieving strong coordination. The linear baseline, which processes Passenger observations independently, achieves moderate returns of 22.0 but lacks mechanisms to exploit inter-agent dependencies. The encoder-only model performs slightly worse, suggesting that although shared attention over local observations provides some coordination signal, the absence of cross-attention limits its effectiveness. The full encoder–decoder transformer yields the highest mean performance of 27.6, a 25.45% increase over the baseline. Combining global attention with cross-attention between Passenger tokens enables richer coordination and more effective role assignment. Finally, the max action variant performs comparably to the full model, but with slightly reduced mean performance and higher variance, reflecting that enforcing single-role behaviors can work in some environments but sacrifices the flexibility needed to adapt across diverse task configurations.

### 4.3 MCAPS performance against fixed team specializations

**FIGURE 6:**
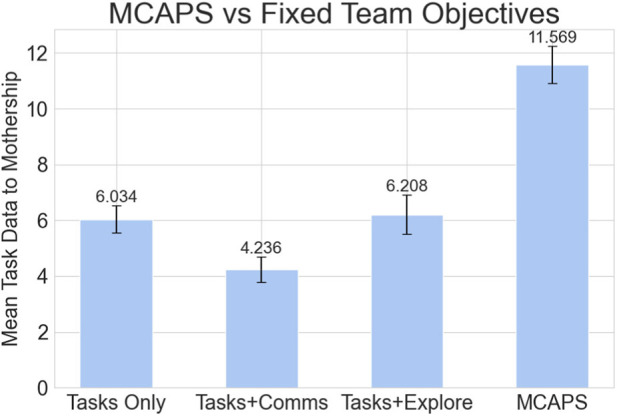
Mean task data returned to Mothership over evaluation episodes for Passenger teams with fixed objective configurations and using MCAPS. These results show that the coordinated specializations learned and assigned by the Mothership’s policy outperform rigid, single-objective Passenger team specializations.

Here, we evaluate MCAPS’s performance against teams with fixed, manually defined specializations. We evaluate against a team, where each Passenger uses only the nearest task objective (Tasks Only), as well as split teams, where half of the Passengers target tasks, while the other half support communications with the neediest communication objective (Tasks + Comms) or explore with the nearest frontier objective (Tasks + Explore). These objectives—completing tasks, relaying communications, and exploring the environment—are all crucial for maximizing data collection in the simulation domain. We evaluate each method in 30 identical, randomly generated configurations and report the mean and standard error of the mean of the data from each completed task sent to the Mothership in each episode in Figure 6. The same 30 environment seeds are used for all methods. Fixed-objective baselines are deterministic given fixed environments; MCAPS results reflect variability from stochastic Passenger planning rollouts.

The results in Figure 6 highlight MCAPS’s ability to provide superior team behavior coordination over fixed per-robot objectives. On average, a team of passengers coordinated using MCAPS returned 86.3% more task data to the Mothership than the next-best fixed-objective team (Tasks + Explore). The purely greedy Tasks Only team performs competitively among the fixed-objective baselines, yet still collects 47.3% less task value than MCAP. This confirms that MCAPS has successfully learned to deviate from greedy behaviors to maximize long-term rewards. We also add that MCAPS has access to additional candidate objective functions (beyond approaching tasks, needy communication points, and frontiers) compared to the fixed, single-objective tests. These additional objectives, combined with the flexibility offered by Passengers’ scalarized objective functions, provide additional avenues for MCAPS to exploit to enhance team coordination.

Overall, these results indicate that the MCAPS coordination policy learns to effectively combine specialization vectors for each Passenger’s candidate objectives to maximize performance in our simulation environment.

#### 4.3.1 Hardware demonstration

**FIGURE 7:**
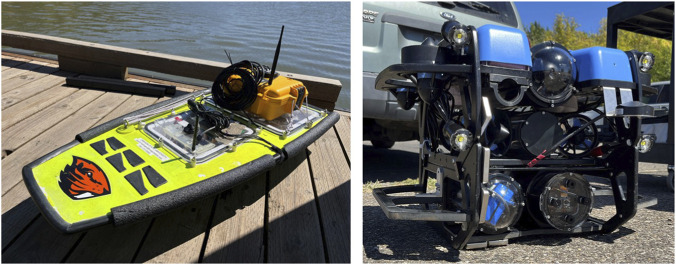
Platypus Lutra USV (left) and Blue Robotics BlueROV (right) used as Passenger robots in MCAPS hardware trials.

Finally, we validate MCAPS in hardware deployments at Fern Ridge Lake in Veneta, Oregon. In our experiments, the lake acts as a surrogate for the under-ice environment and provides an important step toward under-ice deployments. In these tests, the Platypus Lutra USV and Blue Robotics BlueROV Remotely Operated Vehicle (ROV)—shown in Figure 7—act as Passenger robots, while a shore station laptop (Dell XPS15) coordinates their specializations as the Mothership. The ROV communicated with the Mothership base station via a tethered Ethernet connection, while the USV communicated over short-range WiFi; no packet loss was observed for either link during the trials. The USV was localized via GPS, while the ROV used an ultra-short baseline (USBL) acoustic positioning system to localize relative to the base station, serving as a proxy for the localization challenges of an under-ice environment.

**FIGURE 8:**
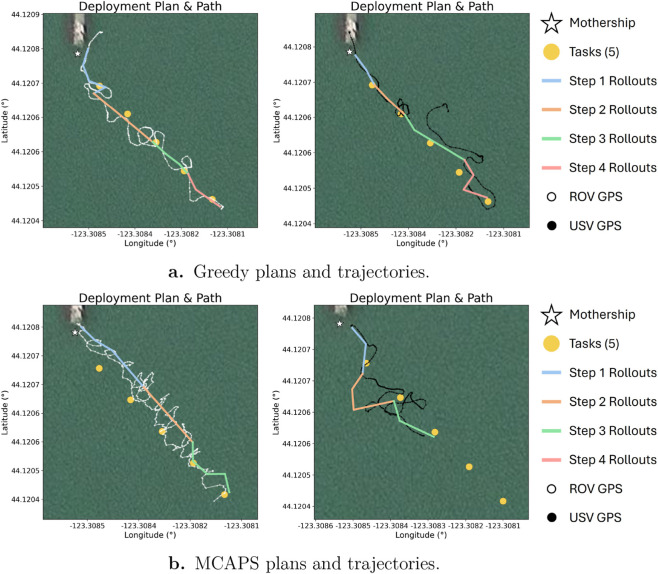
**(a)** Planned and executed trajectories for ROV (left) and USV (right) using greedy Task Only objectives. **(b)** Planned and executed trajectories for ROV (left) and USV (right) using MCAPS coordination. The coordinated trajectories show that the USV completes near- and mid-range tasks, while the ROV completes long-range tasks.

We provide the Passengers with sets of straight-line data collection task waypoints, inspired by underwater glider data collection missions (Zhao et al., 2021; Zhou et al., 2025). We equip the system with the MCAPS algorithm to observe the team’s ability to efficiently complete tasks. Our deployments follow the MCAPS routine: 1) the Mothership base station computes and sends specialization vectors to each Passenger; 2) Passengers create and execute specialized local plans; 3) Passengers send location-stamped observation packets to the Mothership base station from their planned endpoints; and 4) repeat. As a comparison, we provide each Passenger with the greedy “Tasks Only” behaviors discussed in the previous section. Figure 8 shows each robot’s planned paths and executed trajectories for the greedy approach (Figure 8a) and with coordinated specializations (Figure 8b).

We conducted one successful trial of each configuration—greedy Tasks Only and MCAPS—during our lake deployment. For MCAPS, the Mothership completed a policy inference and specialization vector transmission step at the start of each of the three planning cycles required by MCAPS (compared to four for the greedy baseline), with Passengers executing local plans between updates. The greedy approach observes both robots moving together from one task to the next. This behavior is suboptimal as the value obtained from visiting a task decreases with the distance that a message containing that task’s data must travel. As a result, the greedy approach transmits no data from tasks positioned far from the Mothership. Meanwhile, the coordinated trajectories observe the ROV move towards long-range tasks, while the USV executes short- and mid-range tasks. This behavior not only completes all tasks in one less planning cycle but also stations the USV as a midpoint between the ROV and the base station, where it best serves to relay task information and maximize the overall team reward from visiting distant tasks.

These hardware evaluations demonstrate the MCAPS algorithm’s ability to coordinate a real-world multi-robot deployment. Compared to a greedy baseline, the Mothership’s policy effectively adapts each Passenger’s specializations to visit each task waypoint more efficiently.

## 5 Discussion

### 5.1 Limitations

The experimental validation covers simulated underwater environments and a field deployment at an inland lake designed to reflect the coordination and communication challenges that motivate this work. Three constraints specific to actual under-ice environments are not captured and warrant acknowledgment.

1. Vehicle irrecoverability: The lake trials allowed monitoring and recovery without the risk of loss under ice cover. True under-ice deployment prevents surfacing, substantially increasing the cost and risk of vehicle loss; robustness under these irrecoverability constraints remains an open question.
2. Navigation drift: The ROV used USBL-based localization, providing a partially realistic navigation scenario compared to open-water GPS, but not the cumulative drift that accumulates over long-range, surface-denied under-ice missions. The impact of navigation uncertainty on coordination quality is not quantified here.
3. Acoustic communication: The field experiments used tethered Ethernet (ROV) and short-range WiFi (USV), which do not replicate the latency and bandwidth limits of acoustic modems. We note, however, that MCAPS messaging is compact—specialization vectors are O -dimensional floating-point arrays, and observation packets contain sparse < 100-byte regional feature summaries—consistent with data rates achievable by commercial acoustic modems, provided that exchanges are limited to Passenger reporting intervals rather than high-frequency updates.

These limitations do not undermine the generality of the coordination algorithms themselves, but they indicate that full validation in an actual under-ice environment remains a necessary future step before deployment.

### 5.2 Future work

Several promising directions remain to improve our proposed coordination frameworks. First, the current MEPO algorithm does not explicitly combine Mothership-generated plans with locally generated Passenger plans, often resulting in inefficiencies or duplicate solutions. A natural extension would be to investigate methods for merging global and local plans in a more principled manner, for example, by operating over smaller plan segments or by jointly optimizing hierarchical plans across multiple levels of abstraction. Such approaches could reduce repeat or conflicting planning solutions, leading to tighter coordination. Second, although our reward formulation for training the MACPS policy enables sparse, long-horizon training, the resulting signal remains noisy, which can limit policy refinement. Future work could explore alternatives to the reward formulation by incorporating novelty-seeking or intrinsic motivation signals (Pathak et al., 2017; Weltevrede et al., 2024), which can mitigate sparsity and provide smoother gradients for fine-tuned coordination behaviors. These approaches may also encourage a wider variety of emergent role assignments without requiring extensive reward shaping.

### 5.3 Conclusion

The opportunities that Mothership–Passenger systems provide for exploring isolated, dangerous environments require coordination approaches that can account for environmental dynamics and uncertainty alongside complex and changing team priorities. In this study, we have provided a problem formulation for the stochastic multi-agent orienteering problem and presented MEPO, an effective method for merging the benefits of centralized planning on a Mothership with decentralized planning at the Passenger level, to solve this problem. We have also developed MCAPS, a hierarchical approach that coordinates interconnected Passenger robot behaviors as the needs of the team change, and validated this method in real-world hardware experiments. These algorithms advance scalable multi-robot coordination for communication-constrained underwater environments and lay a principled foundation for future deployments in isolated, data-rich environments, such as under-ice ocean regions.

## Data Availability

The datasets presented in this study can be found in online repositories. The names of the repository/repositories and accession number(s) can be found at: https://github.com/natbut/mr-specializations, https://github.com/natbut/mcts-smop.
